# Detection of Anammox Activity and 16S rRNA Genes in Ravine Paddy Field Soil

**DOI:** 10.1264/jsme2.ME11330

**Published:** 2012-02-22

**Authors:** Yoshinori Sato, Hiroyuki Ohta, Takao Yamagishi, Yong Guo, Tomoyasu Nishizawa, M. Habibur Rahman, Hisao Kuroda, Task Kato, Masanori Saito, Ikuo Yoshinaga, Kazuyuki Inubushi, Yuichi Suwa

**Affiliations:** 1 Institute for Global Change Adaptation Science, Ibaraki University, Mito, Ibaraki 310–8512, Japan; 2 Ibaraki University College of Agriculture, 3–21–1 Chuou, Ami-machi, Ibaraki 300–0393, Japan; 3 Research Institute for Environmental Management Technology, National Institute of Advanced Industrial Science and Technology, 16–1 Onogawa, Tsukuba, Ibaraki 305–8569, Japan; 4 Field Science Center, Graduate School of Agricultural Science, Tohoku University, Ohsaki, Miyagi 989–6711, Japan; 5 Laboratory of Marine Microbiology, Division of Applied Biosciences, Graduate School of Agriculture, Kyoto University, Kyoto 606–8502, Japan; 6 Graduate School of Horticulture, Chiba University, 648 Matsudo, Chiba 271–8510, Japan

**Keywords:** anaerobic ammonium oxidation, ravine paddy field soil, nitrogen removal

## Abstract

An anammox assay involving a ^15^N tracer and gas chromatography-mass spectrometry revealed that the potential anammox activity accounted for 1 to 5% of total N_2_ production in a ravine paddy field, Japan. Among four 4-cm-deep layers, the top layer showed the highest activity. Clone libraries showed that the DNA in the top layer contained sequences related to those of *Candidatus* ‘Brocadia fulgida’, *Ca.* ‘B. anammoxidans’, and *Ca.* ‘Kuenenia stuttgartiensis’. These results suggest that a specific population of anammox bacteria was present in paddy soils, although a small part of dinitrogen gas was emitted from the soil via anammox.

Anaerobic ammonia oxidation (anammox) is an important pathway in the microbial nitrogen cycle that allows ammonia to be oxidized by nitrite under anoxic conditions (11). Previous studies have reported the distribution, diversity, and activity of anammox bacteria in several marine ecosystems, and anammox is recognized as an important process in the marine nitrogen cycle (2–5, 8, 13). Recently, it was reported that anammox 16S rRNA gene sequences were detected in marshes, lakeshores, a contaminated porous aquifer, permafrost soil, and agricultural soil, and these sequences showed higher diversity than in marine environments (6). In this study, we focused on the activity and diversity of anammox bacteria in the soil and water above the soil surface in a ravine paddy field, which receives nitrate-contaminated water from vegetable fields on the adjacent plateau and is marsh-like all the year round, making it a suitable niche for anammox bacteria.

The study site was located in Ibaraki prefecture, on the Kanto plains of Japan and has been maintained as an experimental paddy field for studies of nitrate removal for 20 years (7, 12) (Fig. 1). The groundwater pouring into the paddy fields contains a high concentration of nitrate, which is derived from the fertilizers and manure applied to the vegetable fields on the above plateau (7, 12). Samples of surface water, namely water layer overlaying the soil surface, and core soil (sandy loam) were collected from layers located at depths of 0 to 4, 4 to 8, 8 to 12, 12 to 16, and 16 to 20 cm by acrylic resin tubes (25 cm in length, 10 cm in diameter) in May 2008. The samples were placed in sterile polyethylene bags, transported to our laboratory, and stored at 4°C in the dark for a few days. Further soil samples were collected from the surface layer (0 to 8 cm deep) in May 2007 and September 2008 from the same spot and stored at −20°C, before being used for molecular analysis.

Analyses of the inorganic cations (NH_4_^+^) and anions (NO_2_^−^ and NO_3_^−^) in the surface water and soil samples were performed by directly injecting the sample water collected by centrifugation at 15,000 rpm for 10 min into a Shim-pac IC-C1 (Shimadzu, Kyoto, Japan) in an HIC-6A Ion Chromatograph system (Shimadzu), and a TSK-gel IC-Anion-PW (Tosoh, Tokyo, Japan) in an Agilent1100 HPLC system (Agilent Technologies, CA, USA) composed of a G1311A quaternary pump and G1315A diode array detector operating at 210 nm, respectively. There were no significant differences between the chemical properties of any of the soil layers; however, a higher concentration of NO_3_^−^ (530 μM) was detected in the surface water samples (Table 1). This suggests that the surface water of the ravine paddy field receives NO_3_^−^ from the fertilizer and manure applied to the vegetable fields on the above plateau (Fig. 1).

The potential anammox and denitrification activities of soil samples were determined using a ^15^N-tracer technique using gas chromatography-quadrupole mass spectrometry (GC-MS) with the procedures described previously (1, 14), which was based on the method described by Thamdrup and Dalsgaard (13), and includes calculations for necessary calibrations consistent with the theory developed by Spott and Stange (10). Reactive substrates for anammox were added to the vials in the following three combinations to determine the activities of the samples: (i) 0.4 mM unlabeled NH_4_Cl+1 mM Na^15^NO_2_, (ii) 0.4 mM ^15^NH_4_Cl+1 mM unlabeled NaNO_2_, and (iii) 0.4 mM ^15^NH_4_Cl without nitrite. Anaerobic incubation with substrate combinations (i) and (ii), anammox can be detected by the production of ^14^N^15^N (^29^N_2_). Denitrification activity was determined by the production of ^15^N^15^N (^30^N_2_) following anaerobic incubation with combination (i). Substrate combination (iii) was a negative control to examine whether anammox occurs without nitrite. It also served as a negative control to examine oxygen contamination. Experiments for activity determinations were performed in duplicate, and the average values are shown in Table 1. The distributions of anammox and denitrification activity in each layer are shown in Table 1. The highest activity was found in the surface layer (0 to 4 cm depth; 0.06–0.07 μmol N_2_ g-VS^−1^ h^−1^) and decreased gradually from the upper to deeper layers, and no significant anammox activity was detected in the surface water or the deepest layer of soil (16 to 20 cm depth) (Table 1). The high concentrations of NO_2_^−^ in the water in the paddy field are considered to be associated with higher anammox activity in the surface layer soil. Recently, Zhu *et al.*(15) reported that anammox rates at different depths of paddy soil (0 to 70 cm depth) obtained in Southern China with a high load of slurry manure as fertilizer ranged between 2.9 (0 to 10 cm depth) and 0.5 (60 to 70 cm depth) nmol-N g wet soil^−1^ h^−1^. The potential anammox rates determined in our surface layer soil (2.2–2.7 nmol-N_2_ g wet soil^−1^ h^−1^) were in the same range as those in Southern China. The topography shown in Fig. 1 is common in the Kanto plains of Japan. As far as we have examined, anammox activity has been detected in another ravine in Chiba prefecture (data not shown). These findings suggest that the anammox pathway is widely distributed in paddy fields loaded with nitrogen fertilizer.

The contribution of anammox, based upon determined potential activities, was greatest in the upper layer, accounting for 5% of total N_2_ production, and lowest in the deeper layer (1%) (Table 1). The relatively low anammox contributions compared with those of other environments, such as marine continental-shelf sediment (20–80% of total N_2_ production) (4, 13), an anoxic water column in the Golfo Dulce [19–35% of total N_2_ production (3)] and paddy soil in Southern China [4–37% of total N_2_ production (15)], might have been caused by the higher denitrification activity of our paddy soils.

To construct anammox-specific 16S rRNA gene clone libraries, environmental DNA was extracted using ISOIL for Beads Beating (Nippon Gene, Toyama, Japan) with skimmed milk powder (Wako, Osaka, Japan), according to the method of Nishizawa *et al.*(9). Polymerase chain reaction (PCR) amplification of anammox bacteria-related 16S rRNA genes was performed as described by Amano *et al.*(1). In brief, the following primer sets were used for amplification: S-^*^-Amx-0368-a-A-18 (AMX368F) (5′-TTCGCAATGCCCGA AAGG-3′) and S-^*^-BS-820-a-A-22 (AMX820R). The PCR cycle consisted of denaturing at 95°C for 4 min, annealing at 56°C for 30 sec, and elongation at 72°C for 1 min. The reaction was performed for 30 cycles, and final extension was performed at 72°C for 7 min. The purified PCR products (approximately 450 bp) were ligated into the pT7 Blue T-Vector (Novagen, Madison, WI, USA) using the DNA ligation kit Ver. 2 (Takara Shuzo, Japan) and transformed into *E. coli* DH5α cells (Takara Bio, Otsu, Japan), according to the manufacturer’s instructions. Recombinant clones were employed for the sequencing of both strands using the Big Dye Terminator v3.1 Cycle reaction Kit (Applied Biosystems, Foster City, CA, USA) and a 3130x ABI Prism DNA sequencer (Applied Biosystems).

A total of 120 insert sequences were obtained from the surface layers (0 to 8 cm depth) in May 2007 (34 sequences) and September 2008 (86 sequences). These sequences were grouped into 26 unique operational taxonomic units (OTU) using the web-based bioinformatics platform FastGroupII with a 99% sequence similarity cutoff value. Representative clone sequences of each OTU and their relationships with known anammox bacterial sequences are shown in Supplementary Table 1. A neighbor-joining tree was constructed using the representative clone sequences of each OTU and their evolutionary distance matrix (Fig. 2). In this tree, all of the representative clone sequences were phylogenetically distributed within the anammox-related bacterial lineage (Brocadiales) in the order Planctomycetes. The 46% of total clones sequenced in this study (amx07Y-15 and amx08Y-26; 54/120) were affiliated with *Candidatus* ‘Brocadia fulgida’ with ≥97% sequence similarity. Interestingly, the 16S rRNA gene sequences identical to this clone were recovered from the sediment of a river mouth estuary (1) and a freshwater lake (14), in which evident anammox activities were observed. Especially in the anammox “hot spot” in Lake Kitaura, the nearest lake to the paddy field in this study, 16S rRNA genes closely related to amx07Y-15 and amx08Y-26 were dominantly recovered in the clone library analysis, suggesting that a similar anammox bacterial population is present both in paddy field and freshwater lake sediment. The remaining clone sequences were phylogenetically related to *Ca.* ‘Kuenenia stuttgartiensis’, *Ca.* ‘Anammoxoglobus propionicus’, *Ca.* ‘Brocadia anammoxidans’, or *Ca.* ‘B. fulgida’; however, these OTU formed their own clusters and displayed low similarities to known anammox-related bacteria (94–96%). The relatively higher diversity of anammox bacteria observed in the paddy field in our study and in Southern China (15) and various soil ecosystems (6) than other ecosystems, such as marine environments, may reflect that the soil ecosystem supplies a larger variety of anammox niches (6, 15).

In conclusion, this study detected anammox activity in a paddy field, which was attributed to *Ca.* ‘Brocadia’ and ‘Kuenenia’-related anammox bacteria. Anammox bacteria makes a small contribution to dinitrogen emission from constantly submerged paddy soils in ravine environments. Further studies on the seasonal changes in the distribution, diversity, and activity of anammox bacteria are needed to assess the quantitative contribution of the anammox process to nitrogen removal in paddy fields.

## Supplementary Material



## Figures and Tables

**Fig. 1:**
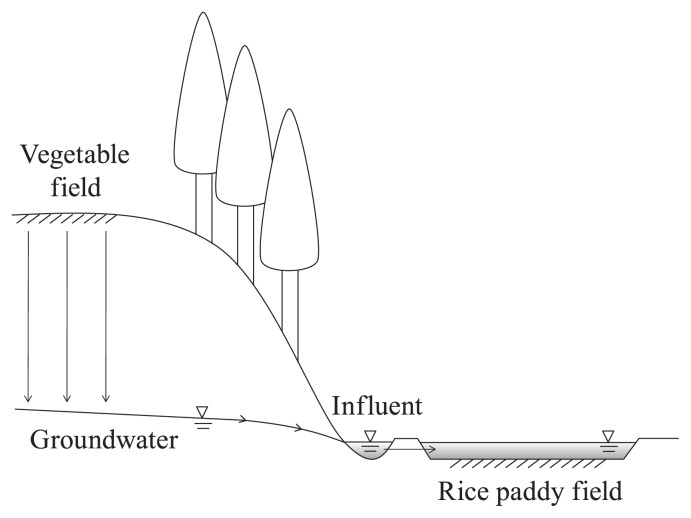
A rough sketch of the topographic profile of the ravine paddy field. The difference in elevation between the groundwater table and the surface of the vegetable field is about 7 meters. The distance between the vegetable field and influent is about 50 meters.

**Fig. 2:**
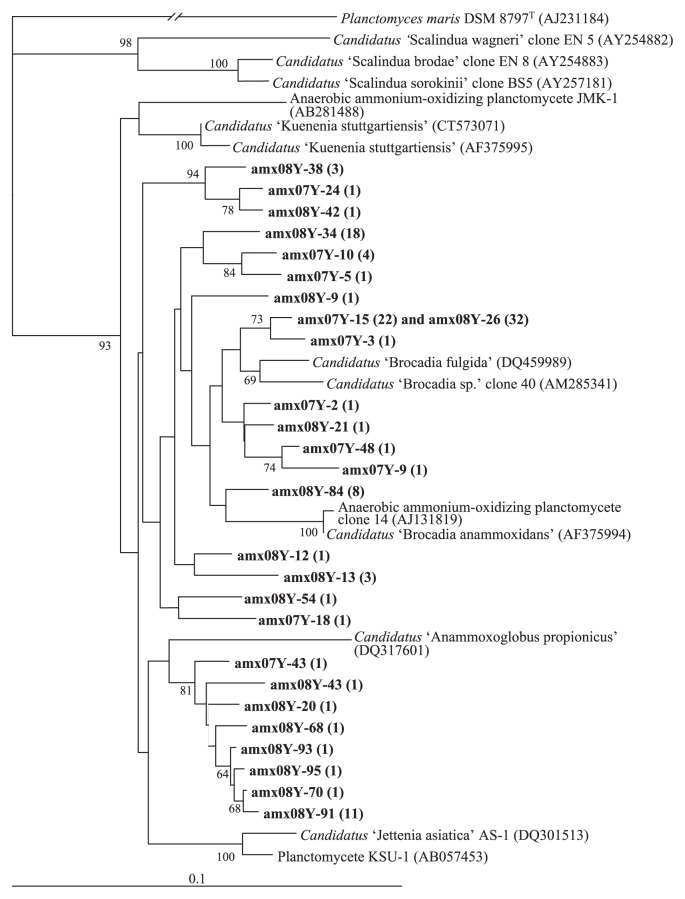
Neighbor-joining tree based on 16S rRNA gene sequences, showing the phylogenetic relationship between anammox-related sequences collected from a ravine paddy field (shown in bold) and known anammox bacterial sequences found in the order *Planctomycetales*. Values along the branches indicate bootstrap percentages of >60%, based on 1,000 resamplings. Bar, 0.1 substitutions per nucleotide position.

**Table 1:** Concentrations of ammonium, nitrate, and nitrite, and anammox and denitrification activities in the soil and water of a ravine paddy field

Samples	Concentration (μM)			Anammox activity (μmol N_2_ g-VS^−1^ h^−1^)^a^		Denitrification activity (μmol N_2_ g-VS^−1^ h^−1^)^a^	Relative anammox (%)^b^
	
	NH_4_^+^	NO_2_^−^	NO_3_^−^	^15^NH_4_^+^+NO_3_^−^	NH_4+_+^15^NO_3_^−^		
water	0	3.5	530	N.D.	N.D.	N.D.	N.D.
soil							
0–4 cm	27	0.5	6	0.06	0.07	1.42	5
4–8 cm	21	0.4	7	0.02	0.02	0.62	3
8–12 cm	28	0.4	5	0.001	0.005	0.23	2
12–16 cm	23	1.0	4	0.001	0.004	0.37	1
16–20 cm	35	0.3	3	N.D.	N.D.	N.D.	N.D.

a The experiments were performed in duplicate, and average values are shown.

b The relative anammox ratio of total N_2_ gas production.
