# Supplementary material for: Detection of Anammox Activity and 16S rRNA Genes in Ravine Paddy Field Soil
Source: Microbes Environ. 2012 Feb 22;27(3):316–9. doi: 10.1264/jsme2.ME11330 (PMC4036040; doi:10.1264/jsme2.ME11330)
Supplement: Supplementary file 1 [file 27_316_s1.pdf]

Supplementary Table S1. Sequence similarities between the sequences of known anammox bacteria and clone sequences recovered from the ravine paddy field.

OTU[a]	Representative sequence	Accession no.[b]	No. of clones in library		Known anammox sequences	Accession no.	Similarity[c] %
			May 2007	Sep. 2008			
1	amx08Y-38	AB602697	0	3	*Ca.* ‘Kuenenia stuttgartiensis’	CT573071	94
2	amx07Y-24	AB602650	1	0	*Ca.* ‘Kuenenia stuttgartiensis’	CT573071	94
3	amx08Y-42	AB602700	0	1	*Ca.* ‘Kuenenia stuttgartiensis’	CT573071	94
4	amx08Y-34	AB602693	0	18	*Ca.* ‘Brocadia fulgida’	DQ459989	95
					Ca. ‘Kuenenia stuttgartiensis’	CT573071	95
5	amx07Y-10	AB602637	4	0	*Ca.* ‘Brocadia fulgida’	DQ459989	95
					Ca. ‘Kuenenia stuttgartiensis’	CT573071	95
6	amx07Y-5	AB602633	1	0	*Ca.* ‘Kuenenia stuttgartiensis’	CT573071	95
7	amx08Y-9	AB602670	0	1	*Ca.* ‘Kuenenia stuttgartiensis’	CT573071	95
					Ca. ‘Brocadia fulgida’	DQ459989	95
					Ca. ‘Brocadia anammoxidans’	AF375994	95
8	amx07Y-15	AB602642	22		*Ca.* ‘Brocadia fulgida’	DQ459989	97
	amx08Y-26	AB602686		32	*Ca.* ‘Brocadia fulgida’	DQ459989	97
9	amx07Y-3	AB602631	1	0	*Ca.* ‘Brocadia fulgida’	DQ459989	97
10	amx07Y-2	AB602630	1	0	*Ca.* ‘Brocadia fulgida’	DQ459989	96
11	amx08Y-21	AB602681	0	1	*Ca.* ‘Brocadia fulgida’	DQ459989	95
					Ca. ‘Brocadia anammoxidans’	AF375994	95
12	amx07Y-48	AB602662	1	0	*Ca.* ‘Brocadia fulgida’	DQ459989	94
					Ca. ‘Kuenenia stuttgartiensis’	CT573071	94
					Ca. ‘Brocadia anammoxidans’	AF375994	94
13	amx07Y-9	AB602636	1	0	*Ca.* ‘Brocadia fulgida’	DQ459989	94
14	amx08Y-84	AB602737	0	8	*Ca.* ‘Brocadia fulgida’	DQ459989	96
					Ca. ‘Brocadia anammoxidans’	AF375994	96
15	amx08Y-12	AB602673	0	1	*Ca.* ‘Kuenenia stuttgartiensis’	CT573071	94
16	amx08Y-13	AB602674	0	3	*Ca.* ‘Kuenenia stuttgartiensis’	CT573071	95
17	amx08Y-54	AB602709	0	1	*Ca.* ‘Kuenenia stuttgartiensis’	CT573071	95
					Ca. ‘Brocadia anammoxidans’	AF375994	95
18	amx07Y-18	AB602645	1	0	*Ca.* ‘Brocadia fulgida’	DQ459989	94
					Ca. ‘Kuenenia stuttgartiensis’	CT573071	94
19	amx07Y-43	AB602658	1	0	*Ca.* ‘Kuenenia stuttgartiensis’	CT573071	95
20	amx08Y-43	AB602701	0	1	*Ca.* ‘Kuenenia stuttgartiensis’	CT573071	95
21	amx08Y-20	AB602680	0	1	*Ca.* ‘Kuenenia stuttgartiensis’	CT573071	95
22	amx08Y-68	AB602722	0	1	*Ca.* ‘Kuenenia stuttgartiensis’	CT573071	94
23	amx08Y-93	AB602743	0	1	*Ca.* ‘Kuenenia stuttgartiensis’	CT573071	95
24	amx08Y-95	AB602745	0	1	*Ca.* ‘Kuenenia stuttgartiensis’	CT573071	94
25	amx08Y-70	AB602724	0	1	*Ca.* ‘Kuenenia stuttgartiensis’	CT573071	95
26	amx08Y-91	AB602741	0	11	*Ca.* ‘Kuenenia stuttgartiensis’	CT573071	95

[a] OTU (operational taxonomic units) were defined by the web-based bioinformatics platform FastGroupII with a 99 % sequence similarity cutoff value.
[b] Nucleotide sequence data reported in this study are available in DDBJ/EMBL/GenBank databases under accession numbers AB602629 to AB602662 for amx07Y-1 to amx07Y-48 and AB602663 to AB602748 for the amx08Y-1 to amx08Y-98, respectively.
[c] Similarity to the closest related database sequence.
